# The Neural Dynamics of Individual Differences in Episodic Autobiographical Memory

**DOI:** 10.1523/ENEURO.0531-19.2020

**Published:** 2020-03-16

**Authors:** Raluca Petrican, Daniela J. Palombo, Signy Sheldon, Brian Levine

**Affiliations:** 1 School of Psychology, Cardiff University, Cardiff, Wales CF10 3AT, UK; 2 Department of Psychology, University of British Columbia, Vancouver, British Columbia V6T 1Z4 Canada; 3 Department of Psychology, McGill University, Montreal, Quebec H3A 1G1, Canada; 4 Rotman Research Institute and Departments of Psychology and Neurology, University of Toronto, Toronto, Ontario M6A 2E1, Canada

**Keywords:** autobiographical memory, dynamic connectivity, episodic memory, functional networks

## Abstract

The ability to mentally travel to specific events from one’s past, dubbed episodic autobiographical memory (E-AM), contributes to adaptive functioning. Nonetheless, the mechanisms underlying its typical interindividual variation remain poorly understood. To address this issue, we capitalize on existing evidence that successful performance on E-AM tasks draws on the ability to visualize past episodes and reinstate their unique spatiotemporal context. Hence, here, we test whether features of the brain’s functional architecture relevant to perceptual versus conceptual processes shape individual differences in both self-rated E-AM and laboratory-based episodic memory (EM) for random visual scene sequences (visual EM). We propose that superior subjective E-AM and visual EM are associated with greater similarity in static neural organization patterns, potentially indicating greater efficiency in switching, between rest and mental states relevant to encoding perceptual information. Complementarily, we postulate that impoverished subjective E-AM and visual EM are linked to dynamic brain organization patterns implying a predisposition towards semanticizing novel perceptual information. Analyses were conducted on resting state and task-based fMRI data from 329 participants (160 women) in the Human Connectome Project (HCP) who completed visual and verbal EM assessments, and an independent gender diverse sample (*N* = 59) who self-rated their E-AM. Interindividual differences in subjective E-AM were linked to the same neural mechanisms underlying visual, but not verbal, EM, in general agreement with the hypothesized static and dynamic brain organization patterns. Our results suggest that higher E-AM entails more efficient processing of temporally extended information sequences, whereas lower E-AM entails more efficient semantic or gist-based processing.

## Significance Statement

The ability to revisit specific events from one’s past is key to identity formation and optimal interpersonal functioning. Nonetheless, the mechanisms underlying its typical interindividual variation are yet to be fully characterized. Here, we provide novel evidence that, among younger adults, dispositional variations in subjective mental time travel draw on the same dynamic and static features of the brain’s architecture that are uniquely implicated in memory for spatiotemporal contexts. Specifically, the subjective sense of being able to revisit one’s past relates to neural mechanisms supporting serial mental operations, whereas difficulties in accessing past experiences may be traced back to a predisposition towards gist-based processing of incoming information

## Introduction

Mental travel to specific past personal events is key to adaptive lifespan development (Fivush, 2011). The contribution of visual imagery to such episodic autobiographical memory (E-AM) feats is well-documented (Greenberg et al., 2005; D’Argembeau and Van, 2006; Daselaar et al., 2008; Vannucci et al., 2016). Nonetheless, a link is yet to be established between dispositional variations in E-AM ability (i.e., ability to recollect majority of previously experienced events) and individual differences in the capacity to visually reconstruct past spatiotemporal contexts in one’s mind. To take a step towards addressing this issue, here, we test whether individual differences in self-rated E-AM draw on some of the neural architecture that supports memory for unique spatiotemporal contexts, specifically, memory for random visual scene sequences (henceforth referred to as visual EM). We thus investigate whether features of the brain’s static and dynamic functional architecture relevant to conceptual versus perceptual processing similarly predict individual differences in visual EM and subjective E-AM.

Current literature suggests that conceptual/meaning extraction processes foster episodic memory (EM) formation (Griffiths et al., 2019; Renoult et al., 2019; Staresina and Wimber, 2019), although there is little research on their relative contribution as a function of task demands and individual differences in E-AM. For example, semantic processes may facilitate EM for words, specifically, memory for whether a word was presented in a given context or not (henceforth referred to as verbal EM) because their present contextual correlates can be integrated within an existing knowledge base. In contrast, semantic processes are less likely to foster recall of random visual scenes because these are less readily mapped onto an existing conceptual template. In the latter scenario, access to high fidelity perceptual representations may be required. Put differently, greater reliance on conceptual as opposed to perceptual features at encoding will be disadvantageous on visual, but not verbal, EM tasks.

We propose that a similar relationship between conceptual and perceptual processes may underlie individual differences in subjective E-AM. Our conjecture is based on theory that distinguishes the state of awareness linked to retrieving conceptual information versus re-experiencing the unique spatiotemporal context of specific past events (noetic vs autonoetic consciousness; Tulving, 1985, 2002). Thus, we argue that individuals who claim superior E-AM are those who show relatively weaker reliance on conceptual, relative to perceptual, processes when encoding new information (including personal events), meaning that they form and subsequently retrieve a perceptually rich memory trace which incorporates relatively few conceptual “sequelae” (i.e., at retrieval, they tend to enter into a state of autonoetic, rather than noetic, consciousness; Tulving, 2002; Gurguryan and Sheldon, 2019).

Using network analysis of functional brain imaging data, we tested two hypotheses focused on how the brain’s stable and time-varying functional architecture relevant to perceptual versus conceptual processes may impact individual differences in visual EM and subjective E-AM. First, with respect to stable brain architecture, we examine whether visual EM and subjective E-AM are linked to neural patterns indicative of more efficient perceptual, but less efficient conceptual, processing [i.e., increased (for perceptual)/reduced (for conceptual) similarity in neural connectivity patterns between rest and the respective task; Neubauer and Fink, 2009; Gold et al., 2013; Heinzel et al., 2014; Schultz and Cole, 2016; Petrican and Levine, 2018].

Second, with respect to dynamic organization, we probe whether superior visual EM and subjective E-AM are both associated with fewer spontaneous (i.e., resting state) transitions from perceptual to meaning extraction mental states. We view a higher number of such transitions as indicating a predisposition towards mapping novel perceptual information onto existing conceptual templates (i.e., preferential reliance on semantic features when encoding new information), which, as argued above, would interfere with both visual EM and subjective E-AM. Our argument is based on proposals that resting state architecture reflects behavioral history and on recent demonstrations of the correspondence in temporal structure between resting state and task-evoked neural dynamics (Wig et al., 2011; Farooq et al., 2019).

This report is organized as follows. Part 1 focuses on a sample of healthy adults from the Human Connectome Project (HCP) with the goal of testing our proposal regarding the role of perceptual versus conceptual processes in visual versus verbal EM. Part 2 focuses on a separate sample of healthy adults who self-rated their E-AM. Its purpose is to determine whether the functional brain organization patterns uniquely linked to visual EM in part 1 predict dispositional variations in self-reported E-AM.

## Part 1: HCP Sample

### Materials and methods

#### Participants

This sample included 329 unrelated participants, whose data had been released as part of the HCP 1200 subjects data package in March 2017. This sample represented the largest number of participants from the HCP 1200 subjects data release who were unrelated to one another and who had available data on all the behavioral and fMRI assessments of interest.

The majority of participants (*N* = 296) were right-handed. The sample included 169 younger men (51 between 22 and 25, 69 between 26 and 30, and 49 between 31 and 36 years of age) and 160 younger women (50 between 22 and 25, 48 between 26 and 30, and 62 between 31 and 36 years of age). Although age is presented here in the range format, as advocated by the HCP team (for the rationale behind this age reporting strategy in HCP data releases, see Van Essen et al., 2012), all our brain-behavior analyses used participants’ actual age in years, as available in the HCP restricted data release.

All participants were screened for a history of neurologic and psychiatric conditions and use of psychotropic drugs, as well as for physical conditions or bodily implants that may render their participation unsafe. Diagnosis with a mental health disorder and structural abnormalities, as revealed by the MRI structural scans, were also exclusion criteria. Participants provided informed consent in accordance with the HCP research ethics board.

#### EM: behavioral measures

A visual scene EM task assessed individual differences in the ability to recollect the unique temporal flow associated with perceptually rich information (i.e., temporally ordered visual scenes). A verbal EM task gauged dispositional variations in the ability to recall information likely to draw on the existing knowledge base (words). Performance on the two EM tasks was significantly positively correlated, *r*_(327)_ = 0.28, *p* = 0.0001. Only the population-normed scores were available for the visual EM task. Nonetheless, scores on the visual and verbal EM tasks showed comparable coefficients of variation [0.082 (verbal EM) vs 0.115 (visual EM)], rendering it unlikely that the observed results were due to restricted range in the verbal EM scores.

##### Visual scene sequences

The NIH Toolbox Picture Sequence Memory test, completed on day 2 of the participants’ HCP schedule, was used to assess EM for temporally ordered visual scenes (Barch et al., 2013). Participants were required to recall increasingly lengthier series of illustrated objects and activities presented in a specific order on a computer screen. Sequence length varied from six to 18 pictures. Participants were given credit for each pair of adjacent pictures correctly recalled up to the maximum value for each sequence, which was one less than sequence length.

##### Verbal

Form A of the Penn Word Memory test (Gur et al., 2001a,b, 2010), a non-NIH Toolbox measure, was completed on day 1 of the participants’ HCP schedule and was used to measure participants’ verbal EM abilities. Participants were presented with 20 words and asked to memorize them for a subsequent test. On the recall trials, they were shown the 20 previously learned words together with 20 new words matched on memory-related characteristics. Participants had to decide whether they had previously seen the word by selecting among the following response options: “definitely yes,” “probably yes,” “probably no,” and “definitely no.”

#### fMRI tasks

The tasks described below were selected with an eye towards ensuring a representative repertoire of spontaneous neurocognitive states likely to be observed during rest. We reasoned that this sampling strategy would help us identify our hypothesis-relevant neurocognitive states with greater accuracy (e.g., in a comparison involving only the perceptual and semantic processing conditions, a predominantly motor state could be mis-classified as reflecting perceptual processing just because of its greater similarity with the perceptual, rather than the semantic, processing state). This is why we included tasks not directly linked to our hypotheses (e.g., the motor task), but which captured mental states highly likely to occur in the scanner (i.e., mental states relevant to body movement).

##### Perceptual processing and online maintenance

As a measure of their ability to process perceptual information and temporarily keep in their minds representations based on rich percepts, participants completed two runs of an n-back task, which included as targets four categories of stimuli: faces, places, tools and body parts. In the present report, we focused on the zero-back condition, in which a stimulus was presented at the beginning of each block and the participants had to respond “target” whenever the respective stimulus was encountered during the block. We considered this task condition, which is analogous to a delayed match to sample procedure, to best exemplify basic processing of perceptual information, including the creation of the relevant mental images. Each run of the zero-back task encompassed four task blocks (27.5 s each), with each comprising all four stimulus categories, presented in separate blocks. Each block began with the 2.5-s presentation of a cue indicating task type and, for the 0-back task only, target stimulus, followed by 10 trials of 2.5 s each (2-s stimulus presentation and 500-ms interstimulus interval) for a total block duration of 27.5 s (Barch et al., 2013).

The two-back condition, which assesses both perceptual processing and updating of online mental contents, was not included in the present report because preliminary classifier analyses revealed that, in individual-to-group mappings of task architecture, the two-back condition could not be reliably differentiated from the zero-back condition. Instead, the serial math task, described below, was employed as a measure of the participants’ ability to manipulate online mental contents.

##### Meaning extraction and manipulation of online mental representations

Brief fables were employed to assess meaning extraction from rich narrative information. Temporally extended manipulation of mental representations was assessed with a math task (serial arithmetic operations). Participants thus completed two runs of a task, adapted from Binder et al. (2011), in which aural presentation of brief stories alternates with aural presentation of math problems. On each run, participants are presented with four story and four math blocks, which are matched in duration. On the story blocks, participants are presented with short adaptations of Aesop’s fables (five to nine sentences), which involve animal and human characters interacting in easily understandable social situations. Subsequently, participants are required to answer a two-alternative forced choice question, which tests their understanding of the story topic. On the math blocks, participants are asked to solve serial addition and subtraction problems. Each series of arithmetic operations (e.g., “four plus twelve minus two plus nine”) ends with the word “equals,” followed by two alternatives (e.g., “thirty-two or twenty-three”). The math task is adapted on an individual basis, so that a similar level of difficulty is maintained across subjects. The story task was designed to tap participants’ ability to extract meaning from incoming perceptual information (i.e., aurally presented stories; Binder et al., 2011). Complementarily, the math task was designed to gauge the participants’ ability to engage in similarly effortful, temporally extended cognitive processes (i.e., aurally presented arithmetic operations), which, however, do not involve meaning extraction processes (Binder et al., 2011). The aforementioned dissociation between the cognitive processes hypothesized to be underlying performance on the story versus the math task is supported by their associated brain activation patterns, as reported in the initial study (Binder et al., 2011) and in the HCP sample (Barch et al., 2013).

##### Motor processing

This task was included in order to account for mental states relevant to actual and/or planned/desired movement, which was expected to occur in the scanner, including movement pertaining to ongoing mind wandering. It was adapted from the one developed by Buckner and colleagues (Buckner et al., 2011; Yeo et al., 2011). In response to visual cues, participants are required to tap their left or right fingers, squeeze their left or right toes, or move their tongue. Each block, corresponding to a movement type, lasts 12 s (10 movements) and is preceded by a 3-s cue. In each of the two task runs, there are two tongue, four finger (two left, two right) and four toe (two left, two right) movement blocks, respectively, as well as three 15-s fixation blocks.

#### fMRI data acquisition

Images were acquired with a customized Siemens 3T Connectome Skyra scanner housed at Washington University in St. Louis (32-channel coil). Pulse and respiration were measured during scanning. T1-weighted anatomic scans were acquired with a 3D MP-RAGE sequence (TR = 2400 ms, TE = 2.14 ms, FOV = 224 mm, 320 × 320 matrix, 256 slices of 0.7-mm isotropic voxels). The high-resolution structural scan preceded the acquisition of functional scans.

Functional images were acquired with a multiband EPI sequence (TR = 720 ms, TE = 33.1 ms, flip angle = 52°, FOV = 208 mm, 104 × 90 matrix, 72 slices of 2 × 2-mm in-plane resolution, 2 mm thick, no gap). For each task, two runs of equal duration were obtained, one collected with a L-R, and the other, with a R-L, EPI phase coding sequence. For rest, four different scans were acquired in two different sessions (two collected with a L-R and two collected with R-L EPI phase coding sequence). In the present study, we used the L-R and R-L resting state scans collected from both sessions (i.e., four runs in total). The length of one run (in minutes) was as follows: 14:33 (rest), 5:01 (perceptual processing), 3:57 (story/math), and 3:34 (motor). Details on the duration of each resting state epoch and task condition, used in the connectivity analyses, are included in the section on fMRI data analysis.

Individual L-R and R-L scans exhibit distinct regions of complete signal loss, but it has been verified that the preprocessed datasets are anatomically well-aligned with one another, even in areas of complete signal loss (Smith et al., 2013). Because it is only the dropout that differs between the two scan types, it has been recommended that connectivity analyses based on HCP data aggregate the respective metrics from the LR and RL scans (Smith et al., 2013). Consequently, in the present report, we concatenated the LR and RL runs for rest and each task (for further details on the concatenation of the resting state scans, see below, fMRI data analysis).

#### fMRI data preprocessing

**Figure 1.**
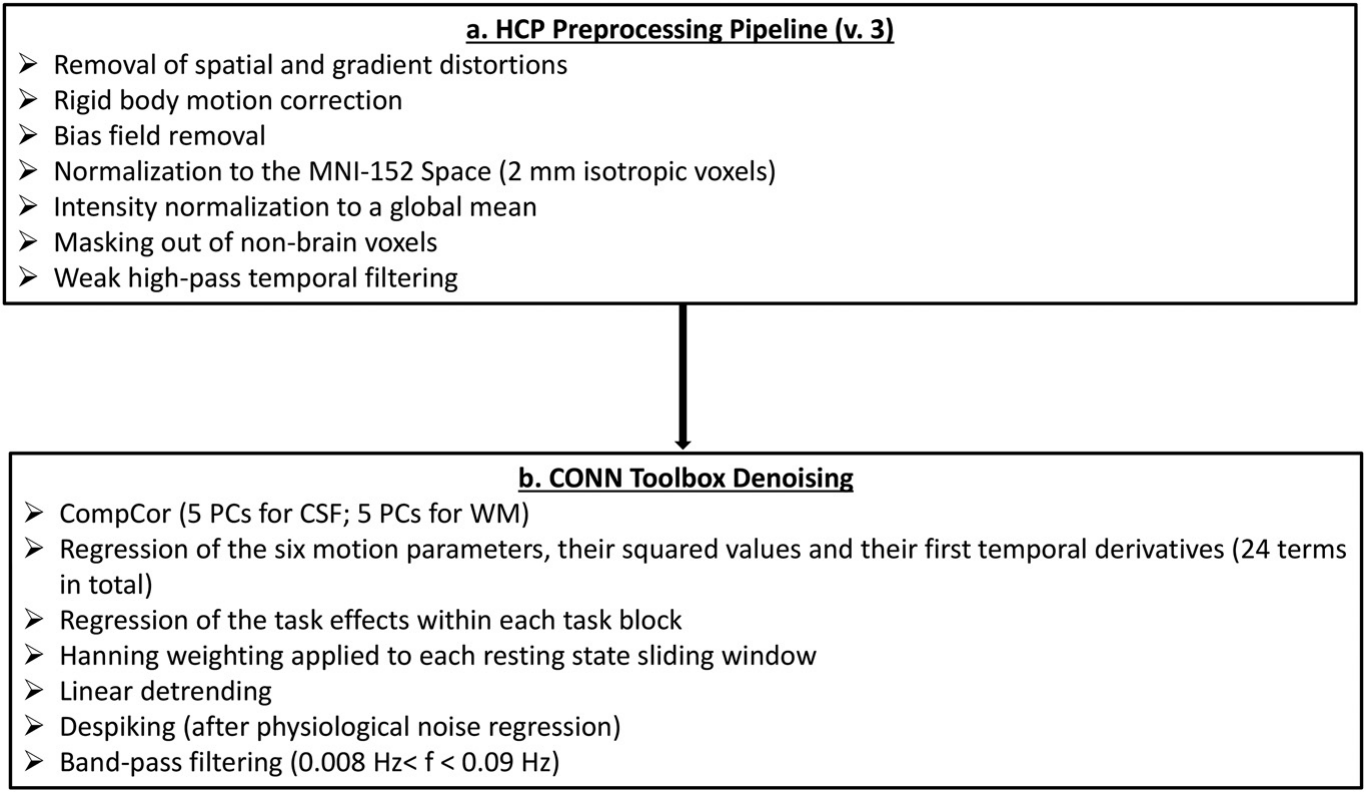
Schematic representation of our preprocessing pipeline.

A schematic representation of our preprocessing pipeline is depicted in Figure 1. In short, the present report used the preprocessed rest and task (i.e., perceptual processing, story/math, and motor processing) data from the HCP 1200 subjects data release. These data all have been preprocessed with version three of the HCP spatial and temporal pipelines (Smith et al., 2013; for specification of preprocessing pipeline version, see http://www.humanconnectome.org/data). Spatial preprocessing involved removal of spatial and gradient distortions, correction for participant movement, bias field removal, spatial normalization to the standard Montreal Neurologic Institute (MNI)-152 template (2-mm isotropic voxels), intensity normalization to a global mean and masking out of non-brain voxels. Subsequent temporal preprocessing steps involved weak high-pass temporal filtering with the goal of removing linear trends in the data.

##### Task fMRI data: regression of condition effects

Our goal was to isolate task-related functional coupling from mere co-activation effects corresponding to the beginning and end of a task block (i.e., two regions that are both activated at the beginning of a task block and de-activated at the end of a task block, although they do not “communicate” with one another throughout the task block). Consequently, following existing guidelines in the literature, we regressed out condition effects from each task block by applying to the BOLD timeseries of each region of interest (ROI) a regressor, obtained by convolving a boxcar task design function with the hemodynamic response function, and its first temporal order derivative (Whitfield-Gabrieli and Nieto-Castanon, 2012; Braun et al., 2015; Vatansever et al., 2015; Westphal et al., 2016). The regression of the condition effects was implemented by using the Denoising step in the CONN toolbox (see paragraph below for additional regressors implemented in this step). All the task-related functional brain organization analyses were conducted only on the task blocks (i.e., the between-task block rest periods were eliminated from the analyses).

##### Task and resting state fMRI data

Because motion can significantly impact functional connectivity measures (Power et al., 2012; Van Dijk et al., 2012), we implemented several additional preprocessing steps to address this potential confound in both the task and resting state data (Fig. 1*B*). In line with prior studies that compared functional brain organization in the task and resting state HCP data (Bolt et al., 2017), these denoising steps were identical for the task and rest data. First, after extracting the BOLD time series from our ROIs (see below, ROI time series), but prior to computing the ROI-to-ROI correlations, we used the Denoising step in the CONN toolbox (version 17c; Whitfield-Gabrieli and Nieto-Castanon, 2012) to apply further physiological and rigid motion corrections. Specifically, linear regression was used to remove from the BOLD time series of each ROI the BOLD time series of the voxels within the MNI-152 white matter and CSF masks, respectively (i.e., the default CONN option of five CompCor-extracted principal components for each, Behzadi et al., 2007), the six realignment parameters, their first-order temporal derivatives and their associated quadratic terms (24 regressors in total; Bolt et al., 2017). For the task fMRI data only, regression of the task effects was applied to the ROI timeseries corresponding to each task block (for details, see above, Task fMRI data: regression of condition effects). The residual BOLD time series for both task and rest were bandpass filtered (0.008 Hz < f < 0.09 Hz), linearly detrended and despiked (all three are default CONN denoising steps). Following these corrections (which did not include global signal regression), an inspection of each subject’s histogram of voxel-to-voxel connectivity values for each scrutinized condition (rest, task) revealed a normal distribution, approximately centered around zero, which would suggest reduced contamination from physiological and motion-related confounds (Whitfield-Gabrieli and Nieto-Castanon, 2012). Nonetheless, in supplementary analyses, accompanying all the brain-behavior tests, we confirmed that all the reported effects were not driven by individual differences in motion, as they remained unchanged after controlling for the average relative (i.e., volume-to-volume) displacement per participant, a widely used motion metric (Power et al., 2012, 2015; Satterthwaite et al., 2013).

#### fMRI data analysis

**Figure 2.**
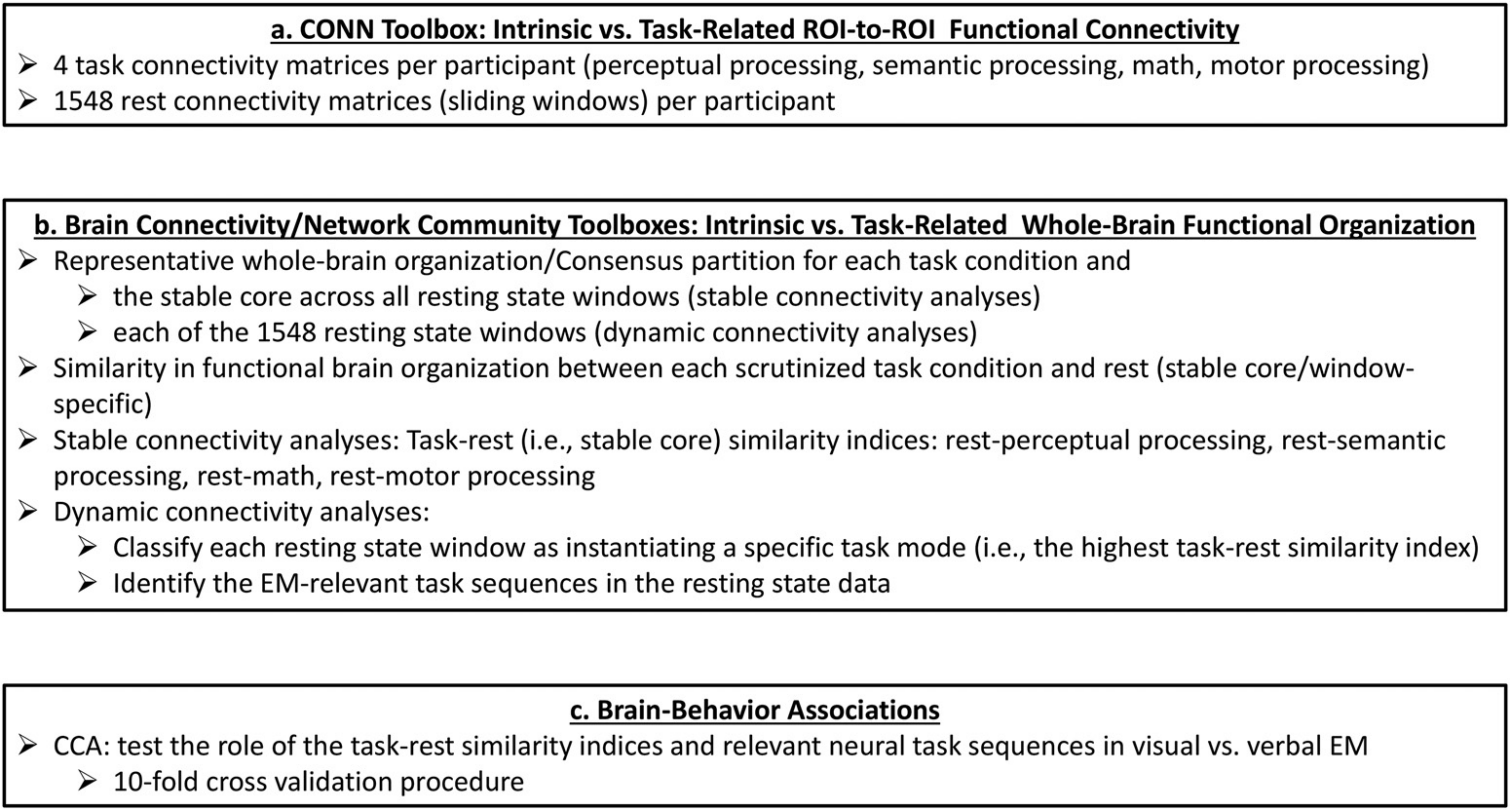
Schematic representation of our analysis pipeline.

A schematic representation of our analysis pipeline is depicted in Figure 2. The specific steps are detailed below.

##### ROI time series

A total of 229 nodes for 10 networks [i.e., default (DMN), frontoparietal (FPC), cingulo-opercular (CON), salience (SAL), dorsal attention (DAN), ventral attention (VAN), somatomotor (SM), subcortical (SUB), auditory (AUD), and visual (VIS)] were defined for each participant as spherical ROIs (radius 5 mm) centered on the coordinates of the regions reported in Power et al. (2011) and assigned network labels corresponding to the graph analyses from this earlier article. The Power et al. (2011) atlas was selected because it was created by taking into account both the task-related activation (derived meta-analytically) and the resting state connectivity patterns of the component voxels for each ROI. Thus, this atlas provided an optimal parcellation scheme for comparing resting state and task-related functional brain architecture.

The ROIs were created in FSL (Smith et al., 2004), using its standard 2-mm isotropic space, with each ROI containing 81 voxels. These template space dimensions were selected because they yielded the most adequate spatial representation of the Power atlas. The 229 ROIs represent a subset of the 264 putative functional areas proposed by Power et al. (2011). The 229 ROIs were selected because, based on Power et al. (2011)’s analyses, they showed relatively unambiguous membership to one of the ten functional networks outlined above.

For each participant, we used the CONN toolbox to compute pairwise bivariate correlations among all 229 ROIs during each scrutinized condition (Fig. 2*A*). Thus, for each participant, we computed 1552 correlation matrices encompassing the pairwise correlations among the 229 ROIs in each condition of interest: rest (1548 matrices), perceptual processing, story, math, and motor processing. For all analyses, the pairwise correlations among all the ROIs were expressed as Fisher’s *z* scores.

Consistent with existing practices aimed at maximizing interpretability of results in neural network studies of individual or group differences (e.g., sex or age, Betzel et al., 2014; Satterthwaite et al., 2015), we used both positive and negative *z* scores to compute the indices of interest for all connectivity analyses. We reasoned that such an approach would be particularly well-justified in our present case since global signal regression, an artefact removal technique that generates negative correlations whose interpretation is still controversial, was not part of our preprocessing pipeline (for further discussion on the validity of the negative correlations obtained with the CONN toolbox, see Whitfield-Gabrieli and Nieto-Castanon, 2012).

##### Task-related connectivity analyses

Pairwise coupling among the 229 ROIs was estimated in CONN, separately for each task condition. The task-relevant connectivity matrices were based on durations ranging from 220 s (i.e., perceptual processing) to ∼240 s (i.e., motor processing). We used such durations because we sought to characterize the stable core of the brain’s functional organization during the task modes under scrutiny (Telesford et al., 2016), which we subsequently compared against the stable and transient aspects of the brain’s functional organization observed during rest.

##### Resting state connectivity analyses

To characterize individual differences in stable and dynamic network structure, we broke down the resting state runs into 1548 windows of 30 s each. This window length was selected in light of prior evidence that it both maximizes detection of individual differences in dynamic network reconfiguration and enables identification of a stable functional core (Leonardi and de Ville, 2015; Telesford et al., 2016; Preti et al., 2017; for similar window sizes in dynamic connectivity analyses of HCP data, see also Chen et al., 2016). Thus, pairwise coupling among the 229 ROIs was estimated in CONN using a sliding window of 30 s in length (∼41 volumes) with a three-TR gap in-between windows and a “hanning weighting” (i.e., greater weight to the scans in the middle of the window relative to the ones at the periphery) applied to all the time points within a window. The use of a hanning weighting was intended to reduce the autocorrelation in the fMRI data series and, thus, maximize the opportunity to detect differences in functional brain organization between adjacent windows. Each window was created so that it would contain only scans acquired with a LR or only scans acquired with a RL encoding sequence. We thus opted to slide the window separately within the LR and RL runs, respectively, in order to eliminate noise that could result from having a window that contained a different proportion of LR and RL scans, which differ with respect to areas of complete signal loss (Smith et al., 2013). This issue was not applicable to the task data, which contained a single window made of an equal number of LR and RL scans.

##### Network-level analyses

All the network-level metrics for both task and rest were computed using the Brain Connectivity Toolbox (BCT; Rubinov and Sporns, 2010) and the Network Community Toolbox (Bassett, D. S.; 2017, November; Network Community Toolbox, retrieved from http://commdetect.weebly.com/), as described below (Fig. 2*B*).

##### Community detection

Rather than being computed directly, the degree to which a network can be fragmented into well-delineated and non-overlapping communities is estimated using optimization algorithms, which sacrifice some degree of accuracy for processing speed (Rubinov and Sporns, 2010). Here, for both task-related and resting state connectivity analyses, the optimal whole-brain division into constituent communities was estimated using a Louvain community detection algorithm implemented in the BCT. This algorithm partitions a network into non-overlapping groups of nodes with the goal of maximizing an objective modularity Q function (Rubinov and Sporns, 2011; Betzel and Bassett, 2017). There are multiple strategies for estimating community structure based on sliding window data, as was the case of our resting state data. Specifically, multilayer modularity algorithms (Mucha et al., 2010; Bassett et al., 2011; Braun et al., 2015) can provide important insights into community dynamics at multiple time scales. Nonetheless, such algorithms require estimation of additional free parameters (e.g., the temporal coupling parameter between two adjacent temporal windows). Since we feared that estimation of the temporal coupling parameter could act as a potential confound when comparing task-related and resting state connectivity results, particularly given the multiple samples included in the analysis, we used the same procedure to estimate community structure independently in each task condition and each of the 1548 resting state time windows (see also Chen et al., 2016), as described below.

For signed networks, such as the ones investigated in our study, optimization of the Q function can be achieved by either placing equal weight on maximizing positive within-module connections and minimizing negative within-module connections or by putting a premium on maximizing positive connections, which have been argued to be of greater biological significance (Rubinov and Sporns, 2011). Although we verified that all the reported results emerge with either formula, for the sake of simplicity and because we agree with their argument regarding the greater importance of positive weights in determining node grouping into communities, we report here the results based on Rubinov and Sporns’s modularity formula (Rubinov and Sporns, 2011; Chen et al., 2016). To account for the near degeneracy of the modularity landscape (Good et al., 2010) and for changes in community structure due to variations in the estimation parameters, for both task-related and resting state connectivity analyses, the community detection algorithm was each initiated 100 times for three values of the spatial resolution parameter, centered around the default value of 1 (Braun et al., 2015; Chen et al., 2016; Betzel and Bassett, 2017).

**Figure 3.**
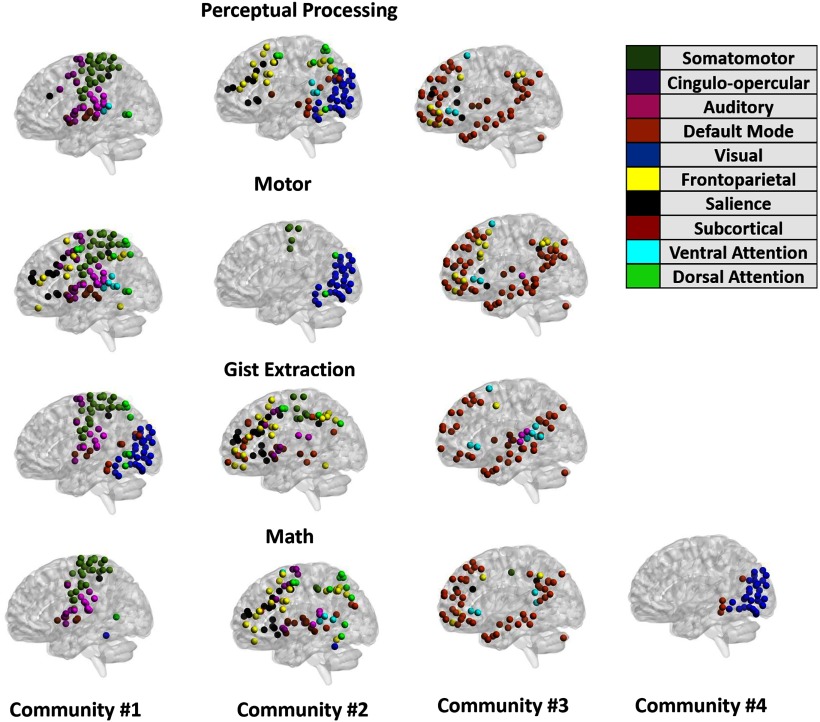
The group-based consensus partitions for each task mode under scrutiny. Network labels are based on Power et al. (2011). The brain networks were visualized with the BrainNet Viewer (http://www.nitrc.org/projects/bnv/; Xia et al., 2013).

Based on the results of these analyses, run separately for each of the three spatial resolution values, a consensus partition (i.e., whole-brain division into constituent communities) was estimated for each participant in each task condition (Lancichinetti and Fortunato, 2012; Bassett et al., 2013). Based on the participant-specific consensus partitions, a group-level consensus partition was estimated for each task mode under scrutiny (see Fig. 3 for the consensus partitions corresponding to each scrutinized task mode at the default value of the spatial resolution parameter and Table 1 for indices of relative similarity in functional brain architecture across the four tasks). For rest, we followed a similar procedure in two steps (Braun et al., 2015). First, we derived a consensus partition for each time window and each participant. Each participant’s window-specific consensus partitions were entered in the analyses involving EM-relevant neural process sequences. Across participants, the average similarity in functional brain organization [i.e., AMI (from 0 no similarity to 1 the two partitions are identical); see section below, “Task-rest similarity in functional brain organization”] between two consecutive windows was 0.61 ± .01. Second, to identify a stable functional core for each participant, we derived a full resting state consensus partition, corresponding to the time scale of the initial sliding windows (∼30 s).

**Table 1:** Average similarity among the group-based task architectures across the three values of the spatial resolution parameter, expressed as the AMI

	Perceptual processing	Semantic processing	Math processing	Motor processing
Perceptual processing	–	0.23	0.53	0.43
Semantic processing	0.23	–	0.32	0.23
Math processing	0.53	0.32	–	0.47
Motor processing	0.43	0.23	0.47	–

The normalized mutual information index ranges from 0 (no similarity between the two partitions) to 1 (perfect similarity between the two partitions).

##### Task-rest similarity in functional brain organization

Each individual’s whole brain functional architecture during rest was compared against the whole-brain organization that typified each scrutinized task mode at the group level. We opted for this approach because we reasoned that it is whole-brain functional architecture, rather than specific ROI-to-ROI connections, that best characterize specific mental states or task modes (i.e., the meaning of specific ROI-to-ROI connections is likely dependent on the whole brain context in which they occur). We thus used the Network Community Toolbox to compute for each participant two types of similarity indices, based on the adjusted normalized mutual information index (AMI), corrected for chance (Vinh et al., 2010). One index type gauged similarity between each participant’s stable functional core, as assessed during rest, and the group-level consensus clustering for each relevant task condition. Specifically, we created four indices reflecting similarity between individual rest and the group-based perceptual processing organization (1), between individual rest and group-based semantic processing organization (2), between individual rest and the group-based math processing organization (3), and between individual rest and group-based motor processing organization (4).

The second index type measured similarity between the group-level consensus clustering for each relevant task condition and each participant’s window-specific functional brain organization. Based on the highest task-rest similarity value, a specific window from a given participant was classified as reflecting primarily one of the four task modes under scrutiny (perceptual processing, semantic processing/math, motor processing). Individuals who spent more resting state windows in a given task mode showed greater similarity between the stable core of their resting state architecture and the respective task mode (*r*s from 0.29 to 0.33, all *p*s* *<* *0.0001), but not the other task modes (all other *r*s* *<* *0.04).

**Figure 4.**
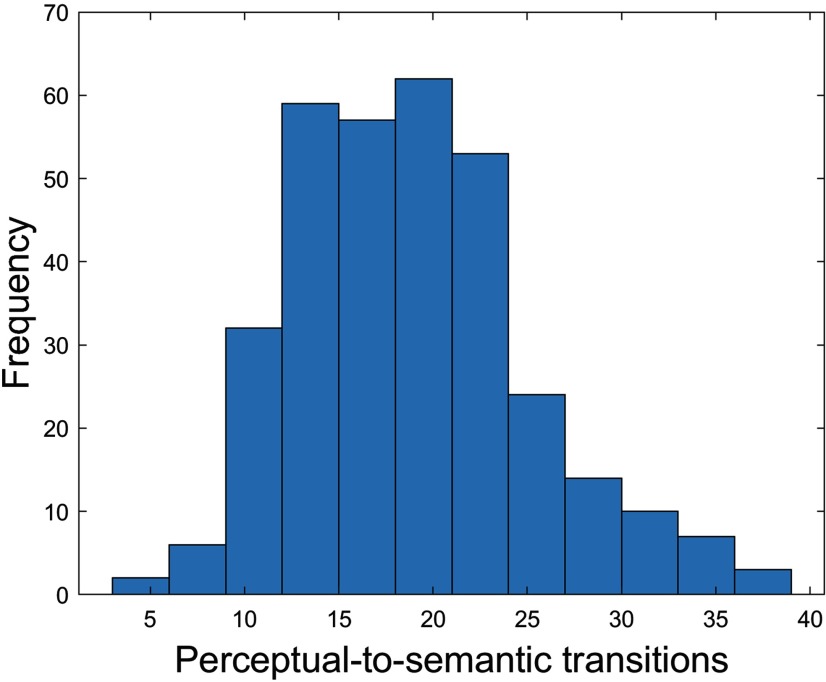
Histogram showing the distribution of our core task switch variable, i.e., number of perceptual-to-semantic processing transitions, in the HCP sample.

For each participant, we counted the number of times a participant expressed the mnemonically relevant sequence [i.e., perceptual (window *n*)-semantic (window *n* + 1) processing] and its counterpart [i.e., semantic (window *n*)-perceptual (window *n* + 1) processing]. Tables 2, 3 contain summary statistics for the similarity indices between the stable core of the brain’s intrinsic architecture and each of the four task modes, as well as summary statistics for the number of windows spent in each task mode and the number of all task mode switches across the full HCP sample. Figure 4 contains a histogram showing the distribution of our core task switch variable, i.e., perceptual-to-semantic processing.

**Table 2:** Average number of task states expressed during rest and average similarity between individual rest and the group-based task architectures across the three values of the spatial resolution parameter in the HCP and the SAM samples

	HCP states (M ± SD)	HCP global AMI (M ± SD)	SAM states (M ± SD)	SAM global AMI (M ± SD)
Perceptual processing	346.80 ± 80.99	0.35 ± 0.09	32.80 ± 12.38	0.11 ± 0.06
Semantic processing	214.35 ± 65.31	0.22 ± 0.06	24.63 ± 10.97	0.07 ± 0.03
Math processing	536.17 ± 88.86	0.33 ± 0.08	66.68 ± 13.03	0.12 ± 0.05
Motor processing	450.68 ± 106.53	0.35 ± 0.08	41.89 ± 15.95	0.12 ± 0.06

M= mean; SD = standard deviation. The HCP data comprises 1548 states, whereas the SAM data comprises 166 states.

**Table 3:** Average number of switches between task states, as observed during rest, across the three values of the spatial resolution parameter in the HCP and the SAM samples

	HCP (M ± SD)	SAM (M ± SD)
Perceptual_Semantic processing	18.75 ± 6.16	2.08 ± 1.46
Perceptual Processing_Math	55.31 ± 10.84	6.28 ± 2.34
Perceptual_Motor processing	41.66 ± 9.52	3.46 ± 1.69
Semantic_Perceptual processing	18.55 ± 6.00	2.36 ± 1.69
Semantic Processing_Math	40.88 ± 11.35	4.77 ± 2.42
Semantic_Motor processing	24.82 ± 7.26	2.46 ± 1.49
Math_Perceptual processing	55.61 ± 11.09	5.99 ± 2.25
Math_Semantic processing	41.06 ± 11.21	4.93 ± 2.79
Math_Motor processing	65.47 ± 12.61	6.88 ± 1.98
Motor_Perceptual processing	41.30 ± 9.27	3.47 ± 1.67
Motor_Semantic processing	24.50 ± 7.16	2.58 ± 1.64
Motor Processing_Math	66.06 ± 12.69	6.79 ± 2.08

M = mean; SD = standard deviation. In the HCP data, there is a maximum of 1544 possible switches, whereas in the SAM data, there is a maximum of 165 switches.

##### Reliability analyses

In line with prior studies on graph theoretical metrics derived from task-based and resting state connectivity patterns (Braun et al., 2012; Cao et al., 2014), we used the intraclass correlation coefficient, ICC (2, 1; Shrout and Fleiss, 1979) to quantify the absolute agreement among the graph theoretical metrics, corresponding to each value of the spatial resolution parameter (see above), computed separately for the day 1 and day 2 resting state sessions. Thus, for each neural index of interest, we entered in the reliability analysis six values, corresponding to day 1, spatial resolution parameter of 0.95, day 1, spatial resolution parameter of 1.00, day 1, spatial resolution parameter of 1.05, day 2, spatial resolution parameter of 0.95, day 2, spatial resolution parameter of 1.00, and day 2, spatial resolution parameter of 1.05.

In line with published criteria (Cicchetti and Sparrow, 1981), as well as prior reliability data for graph metrics derived from task-based and resting state connectivity patterns (Braun et al., 2012; Cao et al., 2014), ICC values ≥0.40 were regarded as reflecting fair to good reliability. Since subject motion can impact such reliability estimates, we present the relevant ICC values, both before and after regressing out subject level average frame-to-frame displacement (please see Preprocessing above for the additional motion effect removal procedures already implemented).

Across the 2 d and three values of the spatial resolution parameter, the task-rest similarity indices relevant to the stable functional core showed ICCs ranging from 0.60 to 0.65 (for data metrics from which the subject-level summary motion metric had been regressed out). For indices from which the subject-level summary motion had not been regressed out, the ICCs ranged from 0.59 to 0.65.

Across the 2 d and three values of the spatial resolution parameter, the number of perceptual-to-semantic transitions showed an ICC of 0.48, irrespective of whether the subject-level summary motion had been regressed out or not. The number of semantic-to-perceptual transitions showed an ICC of 0.48 (ICC of 0.49, if the subject-level summary motion had not been regressed out).

For all the brain-behavior analyses reported below, the stable functional core architecture was estimated based on the resting state data from both days. However, in the reliability analyses reported above, the stable functional core of the resting state architecture was estimated separately for day 1 versus day 2 in order to verify our assumptions (i.e., that there is a person-specific functional core that can be derived from resting state data and that shows some stability across days). The number of state transitions were summed across the two scanning days. In order to obtain more stable estimates of the neural variables of interest, we averaged the homologous indices corresponding to the three values of the spatial resolution parameter for both rest-task similarity metrics (i.e., those relevant to the stable functional core and dynamic neural sequence expression).

##### Validation of the individual to group task-based functional architecture

As outlined above, the analyses herein reported are based on the indices of similarity between group-level task-related and individual-specific resting state functional brain architecture. We opted to do so for two reasons. One was to maximize comparability with the analyses conducted on the survey of autobiographical memory (SAM) sample for which relevant task data were unavailable. The second was to optimize classification of a given resting state window as reflecting primarily one of the four task modes under scrutiny. Specifically, preliminary analyses revealed that in intraindividual comparisons of resting state and task architectures the neural organization within some resting state windows could be equally similar to two or more task modes. Such a pattern of results could emerge even when a window-specific functional brain organization shares the greatest similarity with the key architectural features (i.e., those that are reproduced at the group level) associated with only one task mode. Consequently, we based our analyses on the indices of similarity between the individual-specific resting state architecture and the relevant group-derived task architectures, which yielded unambiguous classification of a resting state window to one of the four task modes under investigation. The group-derived functional task architectures were also used in the comparisons involving the stable functional core within each sliding window.

To verify the validity of our approach, we tested the accuracy of our proposed AMI-based classifier in correctly linking an individual’s task architecture to the corresponding task architecture of a group that did not include the respective individual (e.g., verify that an individual’s functional architecture during story processing is most similar to the group-based story processing functional architecture rather than the group-based perceptual, math or motor processing architectures). To this end, for each task type (perceptual, story, math and motor processing, respectively), we used the ROI-to-ROI correlation matrices, corresponding to each task block, to define through the graph theoretical analyses outlined above (1) a consensus partition corresponding to each block within a given task type (for the motor task only, two task blocks were used), then, based on (1), define (2) a consensus partition characteristic of each task type, which generalizes across different stimulus categories (i.e., the perceptual and motor processing tasks contained stimulus-specific blocks; Barch et al., 2013). The consensus partitions obtained at (2) reflected the brain organization specific to each task type on time scales ranging from approximately 24 s (motor processing) to 27.5 (perceptual processing), hence similar to the time scale used in the resting state dynamic analyses (i.e., 30-s windows). Subsequently, we used a leave-one-subject-out cross validation procedure in which the task architectures of the left-out subject (based on <30 s of data) are evaluated for how similar they are to the group-based stable task architectures derived from the remaining 328 individuals and based on the full task runs of 220–240 s. This procedure was repeated until all participants served as the left-out (“test”) subject. Subsequently, for each individual, we evaluated whether his or her architecture for a specific task condition (based on <30 s of data) showed the greatest similarity to the corresponding group-based architecture (based on 220–240 s of data). Across the four task contexts and the three values of the spatial resolution parameter, our AMI-based classifier had an average accuracy of 66% (±2.10 SE) and a positive predictive value (PPV) of 67% (±2.22 SE).

### Brain-behavior analyses

#### Canonical correlation analysis (CCA)

To characterize the relationship of our neural indices of interest with EM, we used CCA (Hotelling, 1936; Fig. 2*C*) with cross-validation procedures (Hair et al., 1998). CCA is a multivariate technique, which seeks maximal correlations between two sets of variables by creating linear combinations (i.e., canonical variates) from the variables within each set. Recently, CCA has been successfully used to investigate brain-behavior relationships in large datasets (Smith et al., 2015; Tsvetanov et al., 2016; Vatansever et al., 2017). CCA was implemented in MATLAB using the canoncorr module. Task-rest similarity indices for math, perceptual, semantic and motor processing, as well as the number of perceptual-to-semantic and semantic-to-perceptual processing sequences were introduced as brain variables. Age, verbal and visual EM were entered as behavioral variables. Age was introduced in the CCA outlined below because the neural architecture underlying higher-order cognitive functions, including EM, shows protracted development, which extends into the third decade of life (Toga et al., 2006; Lebel et al., 2012; Petrican and Grady, 2017). In order to obtain reliable estimates of canonical loadings (i.e., correlations between the brain or behavioral variables and their corresponding variates), it is generally recommended that CCA be performed on a sample size at least ten times the number of variables in the analysis (Hair et al., 1998), a criterion which was exceeded in all analyses reported below.

The performance of our CCA-derived model of EM was tested by using a 10-fold cross validation procedure. Specifically, the data were broken down into ten folds, all but one containing 30 participants for a total of 329 participants. Discovery CCA was conducted on nine folds of data and the resulting CCA weights were employed to derive predicted values of the brain and behavioral variate in the left-out (test) fold. This procedure was repeated until each of the ten folds served as test data once. The correlation between the predicted brain and behavioral variates across all testing folds was evaluated using a permutation test with 100,000 samples (Smith et al., 2015). To describe the relationship between the behavioral or brain variables and their corresponding variates across all the discovery CCAs, we include canonical loadings (Hair et al., 2009), which reflect the raw correlation between a brain or behavioral variable and its corresponding variate, as well as canonical weights, which indicate the unique contribution of a behavioral or brain variable to its corresponding variate (see also Tsvetanov et al., 2016; Vatansever et al., 2017).

### Code accessibility

The scripts for the graph theoretical analyses outlined above are available in Extended Data 1.

### Results

**Figure 5.**
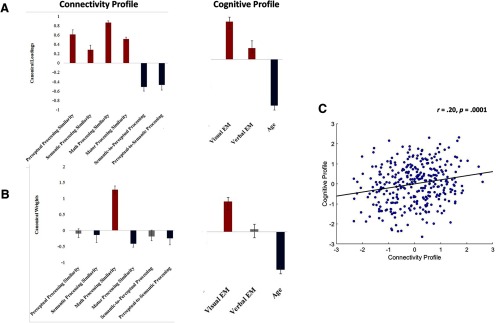
The median canonical loadings (***A***) and the median canonical weights (***B***) of the brain and behavioral variables on their corresponding canonical variate across all discovery CCAs, as well as the scatter plot describing the linear association between the two canonical variates across all the test folds (***C***). In panels ***A***, ***B***, error bars reflect the smallest and largest value, respectively, corresponding to the loading (***A***) or canonical weight (***B***) of each variable on its corresponding variate across all the discovery CCAs. The scatter plot in panel ***C*** is based on standardized variables.

The discovery CCAs detected only one significant mode, which was validated across all test sets (*r = *0.20, *p *=* *0.0001; see Fig. 5*A* for loadings of each connectivity and cognitive variable on its respective canonical variate across all discovery sets; see Fig. 5*B* for standardized coefficients of each connectivity and cognitive variable on its respective canonical variate across all discovery sets; see Fig. 5*C* for the relationship between the predicted brain and behavioral canonical variates across all test sets). The mode identified indicated that younger individuals with superior visual EM demonstrated reduced expression of the perceptual-to-semantic processing sequence, as well as greater similarity between the stable core of the brain’s intrinsic architecture and the functional architecture common to all scrutinized task contexts (Fig. 5*A*), but particularly the math/mental manipulation context (i.e., after accounting for the intercorrelations among the brain variables, the rest-math similarity index showed the strongest association with the brain variate; Fig. 5*B*). Next, we sought to verify that the association between the brain and behavioral variate is not contaminated by demographic factors or extraneous neural variables. To this end, we first created a residual brain variate by regressing out from the original brain variate the number of windows spent in each of the four scrutinized task states and the number of switches between task states not included in the discovery CCA. Subsequently, we conducted a partial correlation analysis, based on 100,000 permutation samples, in which we verified that the association between the original behavioral variate and the aforementioned residual brain variate remained significant (*r* = 0.15, *p *=* *0.006) after controlling for sex, handedness, years of education and average volume-to-volume displacement during rest.

## Part 2: SAM Sample

### Materials and methods

#### Participants

The SAM sample included 59 unrelated, neurologically intact adults [mean age: 23.34 ± 4.90 years (median = 22 years); age range: 18–41 years, 15 males]. The majority of participants (*N* = 50) were right-handed. Data from this sample were also included in Sheldon et al. (2016), but there is no overlap in the analyses documented in the respective paper and the present report.

#### Self-reported memory capacity

Self-reported memory capacity at the trait level was assessed with the 26-item SAM (Palombo et al., 2013). SAM requires participants to rate their E-AM, semantic memory, future thinking, and spatial memory, on a five-point Likert scale (1 = strongly disagree to 5 = agree strongly). In all the analyses herein reported, we used the weighted sum scores derived from the episodic, future, semantic and spatial subscales described below (Palombo et al., 2013).

##### SAM-episodic (eight items)

This subscale gauges participants’ ability to recall specific event and contextual details (e.g., “When I remember events, in general I can recall people, what they looked like, or what they were wearing.”; “When I remember events, in general I can recall objects that were in the environment.”). This subscale was regarded as a measure of trait E-AM.

##### SAM-semantic (six items)

This subscale assesses trait-level differences in the participants’ ability to recall factual information (“I can learn and repeat facts easily, even if I don’t remember where I learned them.”; “After I have met someone once, I easily remember his or her name.”).

##### SAM-future (six items)

This subscale measures trait-level differences in the participants’ ability to imagine specific event and contextual details pertaining to future occurrences (“When I imagine an event in the future, the event generates vivid mental images that are specific in time and place.”; “When I imagine an event in the future, I can imagine how I may feel.”).

##### SAM-spatial (six items)

This subscale evaluates trait-level differences in the participants’ spatial navigation skills (“In general, my ability to navigate is better than most of my family/friends.”; “After I have visited an area, it is easy for me to find my way around the second time I visit.”).

##### Intercorrelations among the SAM memory subscales

The episodic SAM subscale showed a significant positive correlation with the semantic subscale (*r* = 0.38, *p *=* *0.003) and a trending positive association with the future subscale (*r* = 0.23, *p *=* *0.074). No other correlations reached statistical significance (all other *p*s* *>* *0.40).

#### fMRI data acquisition

Images were acquired with a Siemens 3T Trio scanner housed at the Rotman Research Institute (32-channel coil: 35 participants; 12-channel coil: 24 participants). Coil type was introduced as a covariate in all brain-behavior analyses reported below. T1-weighted anatomic scans were acquired with a 3D MP-RAGE sequence (TR = 2000 ms, TE = 2.63 ms, FOV = 256 mm, 256 × 256 matrix, 160 slices of 1-mm isotropic voxels). The high-resolution structural scan preceded the acquisition of functional scans.

Functional images were acquired with a T2*-weighted EPI sequence (TR = 2000 ms, TE = 32 ms, flip angle = 70°, FOV = 200 mm, 64 × 64 matrix, 32 axial slices of 3.1 × 3.1-mm in-plane resolution, 4.5 mm thick, no gap). Acquisition of the resting state scan preceded acquisition of the functional task scans, which are not discussed in this report. During their resting state scan, which lasted ∼5.5 min, participants were asked to allow their minds to wander, while keeping their eyes open and focused on a black fixation cross presented on a white background.

#### fMRI data preprocessing

We performed image processing in SPM12 (Wellcome Department of Imaging Neuroscience). Specifically, we corrected for slice timing differences and rigid body motion (which included unwarping) and spatially normalized the images to the standard MNI-152 template (2-mm isotropic voxels).

Because motion can significantly impact functional connectivity measures (Power et al., 2012; Van Dijk et al., 2012), we used the Denoising step in the CONN toolbox to implement several additional preprocessing steps, which were also applied to the data from the HCP sample, in order to address this potential confound (Fig. 1, step 2). Following these corrections (which did not include global signal regression), an inspection of each subject’s histogram of voxel-to-voxel connectivity values revealed a normal distribution, approximately centered around zero, which would suggest reduced contamination from physiological and motion-related confounds (Whitfield-Gabrieli and Nieto-Castanon, 2012). Nonetheless, same as we did for the HCP data, in supplementary analyses, accompanying all the brain-behavior tests, we confirmed that all the reported effects were not driven by individual differences in motion, as they remained unchanged after controlling for the average relative (i.e., volume-to-volume) displacement per participant, a widely used motion metric (Power et al., 2012, 2015; Satterthwaite et al., 2013).

#### fMRI data analysis

For all analyses, we followed the same steps as the ones outlined for the HCP sample (Fig. 2, steps 1 and 2). Because of the duration of the resting state scan in the SAM sample, all analyses were based on 166 sliding windows with each window being moved in increments of one TR [i.e., 2 s, a duration similar to the one used in the HCP data (3 TRs = 2.16 s)]. All other parameters were identical to the ones used with the HCP data. Across participants and across the three values of the spatial resolution parameter, the average AMI between consecutive windows was 0.63 ± 0.01.

#### Brain-behavior analyses

The goal of these analyses was to test the hypothesis that the neural profile significantly linked to visual EM in the HCP sample would be linked to E-AM abilities, but not the other mnemonic traits assessed by the SAM. To identify the brain variables that make the most reliable contribution to the brain variate linked to visual EM, we conducted a multiple regression analysis across the ten non-overlapping test samples from the HCP. As outcome, we used the standardized value associated with the predicted brain variate score (as derived from the discovery CCAs), from which we regressed out the observed values associated with potential neural confounds (i.e., number of windows spent in each of the four task modes, task mode switches beyond the semantic-to-perceptual and perceptual-to-semantic processing sequences). As predictors, we used the standardized observed values associated with the brain variables of interest (i.e., global similarity indices for perceptual, semantic, math and motor processing, as well as the number of semantic-to-perceptual and perceptual-to-semantic processing sequences). No outliers (i.e., values of 3.29 standard deviations above/below the sample means) were detected among any of these variables. To identify the variables that make a reliable contribution to the aforementioned residual brain variate, we used the bootci function in MATLAB (with default settings and 100,000 bootstrap samples) to obtain 95% confidence intervals (CIs) for each predictor variable. Results of this analysis revealed that only the global perceptual processing-rest similarity index (95% CI = [–0.20; 0.0004]) and expression of the semantic-to-perceptual processing sequence (95% CI = [–0.04; 0.17]) did not make reliable contributions to the residual brain variate.

Hence, based on the results from the multiple regression analysis conducted in the HCP sample (see above), expression of the semantic-to-perceptual processing sequence and similarity between rest and perceptual processing were not included in the computation of the brain variate (i.e., their respective weights were set to zero) in the SAM sample. Instead, these two variables were covaried out from the brain variate because their unreliable contribution to the brain variate was regarded as a potential source of noise, a concern that was rendered salient by the smaller SAM sample (relative to the HCP sample). As in the HCP data, other neural confounds regressed out from the HCP brain variate as well included the number of windows spent in each task mode and number of switches beyond the perceptual-to-semantic processing switch. The resulting residual brain variable was introduced in the correlational analysis described below.

**Figure 6.**
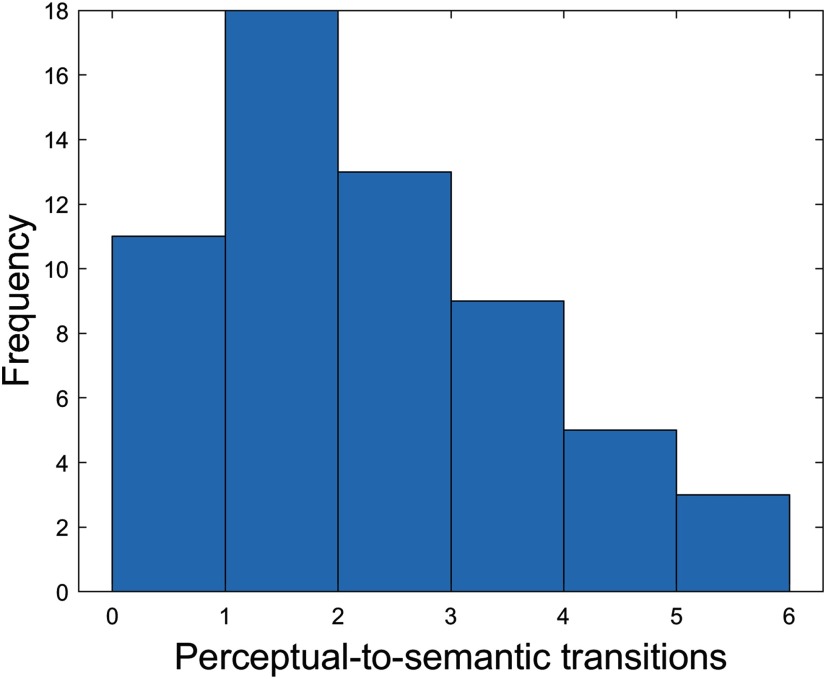
Histogram showing the distribution of our core task switch variable, i.e., number of perceptual-to-semantic processing transitions, in the SAM sample.

Tables 2, 3 contain summary statistics for the similarity indices between the stable core of the brain’s intrinsic architecture and each of the four task modes, as well as summary statistics for the number of windows spent in each task mode and the number of all task mode switches across the full SAM sample. Figure 6 contains a histogram showing the distribution of our core task switch variable, i.e., perceptual-to-semantic processing.

### Results

**Figure 7.**
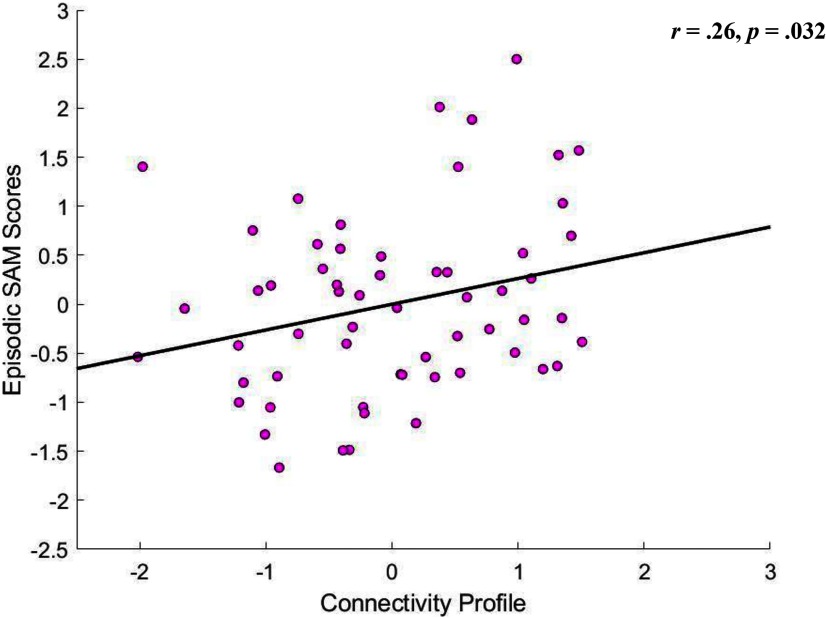
Scatter plot describing the association between episodic SAM scores and the residual brain variate linked to visual EM in the HCP sample (see main text for further details).

Results of a partial correlation analysis, based on 100,000 permutation samples, in which we controlled for scores on the remaining three SAM subscales, as well as age, sex, education, handedness, coil type and head motion, revealed the predicted positive association between episodic SAM scores and expression of the brain pattern linked to visual EM in the HCP sample, *r* = 0.26, *p *=* *0.032 (Fig. 7). As expected, correlational analyses, based on 100,000 permutation samples, showed no similar associations between the neural organization patterns linked to superior visual EM in the HCP sample and scores on the remaining SAM subscales (all *r*s* *<* *0.05, all *p*s* *>* *0.53). Using an on-line calculator for comparing correlation coefficients drawn from the same sample (https://www.psychometrica.de/correlation.html#dependent; Lenhard and Lenhard, 2014), we confirmed that the brain pattern linked to visual EM in the HCP sample was significantly more strongly correlated with the SAM Episodic scores than with the SAM Semantic (*z *=* *2.37, *p* = 0.009) or Spatial (*z *=* *2.01, *p* = 0.022) scores. However, the visual EM-linked brain pattern appeared to be similarly linked to SAM future and SAM episodic (past) scores (*z *=* *1.346, *p* = 0.089), a finding that is compatible with the interpretation that the neural profile herein identified may be broadly relevant to both prospective and retrospective episodic thought.

## Discussion

To date, most neuroscientific investigations on dispositional variations in E-AM have focused on variations in the brain’s structural architecture (Freton et al., 2014; Hodgetts et al., 2017; Hebscher et al., 2018; Palombo et al., 2018a; but see Sheldon et al., 2016). The present study draws on the reportedly key role of visualization in fostering E-AM (Greenberg et al., 2005; D’Argembeau and Van, 2006; Daselaar et al., 2008; Vannucci et al., 2016) to provide novel evidence that spontaneous neural dynamics linked to memory formation processes constitute a common mechanism underlying individual differences in visual EM and subjective E-AM, but not other forms of subjective memory ability (spatial or semantic memory skills). Specifically, we show suggestive evidence that superior performance-based memory for unique spatiotemporal contexts and self-reported E-AM are linked to greater similarity in static functional brain organization, potentially indicating greater efficiency in switching, between rest and a range of goal-directed mental states, as well as a reduced predisposition towards semanticizing perceptual information.

A core motivation of the present research was to investigate whether a competitive relationship between perceptual and conceptual processes shapes individual differences in subjective E-AM and visual EM. Evidence for our proposal was mixed. With respect to the stable core of the brain’s functional architecture, we found some support for our hypothesis that individuals with superior subjective E-AM, as well as those with superior visual EM would show organization patterns suggestive of less efficient semantic processing. Specifically, there was evidence of a reliable unique relationship between superior visual EM and reduced similarity in functional brain organization between rest and the semantic processing mode (Fig. 5*B*), which was replicated with respect to subjective E-AM. In our opinion, these unique effects best capture our hypotheses regarding semantic processing because the raw relationship between the semantic processing variable and the identified brain variate (Fig. 5*A*) is “contaminated” by variance linked to the other brain variables, particularly, variance related to similarity in functional organization between rest and a global task mode, common across all four scrutinized tasks.

Of note, there was no reliable unique association between efficiency in perceptual processing and visual EM (Fig. 5*B*). The lack of a preferential association with visual, rather than verbal, EM is consistent with the interpretation that efficient perceptual processing is core to EM, irrespective of domain and its susceptibility to conceptual contagion (i.e., it contributes to both visual and verbal EM), a finding reported before (Petrican and Levine, 2018).

Clearer support was garnered for our hypothesis relevant to the brain’s dynamic functional architecture (i.e., number of transitions from perceptual to conceptual mental states). As predicted, we found that superior visual EM and subjective E-AM were both linked to less frequent spontaneous transitions between mental states dominated by perceptual processing and those that mainly reflect meaning extraction attempts. We argued that such spontaneous neural dynamics could be interpreted as a predisposition towards mapping perception information onto the relevant conceptual structures. The present results are thus compatible with our proposal that the aforementioned predisposition would impair memory for unique spatiotemporal contexts, as attempts to find a matching conceptual template may distort the mnemonic representation. They are also in line with our hypothesis that a predisposition to semanticize perceptual representations may hinder subjective perceptions of being able to revisit specific past events, potentially because it infringes on the capacity to enter a state of autonoetic consciousness at retrieval (Tulving, 2002).

Beyond our hypotheses, we found that greater efficiency in switching (i.e., reduced functional brain reorganization) from rest to task (Fig. 5*A*) was a hallmark of both superior visual EM and subjective E-AM. With respect to specific task modes, it appears that efficiency in switching to a cognitive mode linked to temporally extended manipulation of mental images (i.e., the math task mode) makes the strongest unique contribution (Fig. 5*B*). Although unpredicted, this effect may reflect the role of serial mental operations in supporting the encoding and reinstatement of spatiotemporal contexts, which are relevant to the presently used visual EM task (Barch et al., 2013) and are generally assumed to also support the subjective sense of being able to revisit the past (Levine et al., 2002; Tulving, 2002; Wheeler and Buckner, 2004).

Interestingly, this neural task mode evidenced the greatest segregation (i.e., the highest number of communities; Fig. 3, math). This pattern likely speaks to its greater processing efficiency and resilience in the face of environmental stressors (Kashtan and Alon, 2005; Kashtan et al., 2007; Braun et al., 2015; Betzel et al., 2016; Sporns and Betzel, 2016). Its associated community structure was compatible with neural organization patterns previously linked to successful episodic learning, such as greater connectivity between the DMN and visual systems, a pattern that is likely relevant to the creation of mental representations based on perceptual information (Fig. 3, community 4; Sheldon et al., 2016). Complementing the aforementioned unique community features of the math task mode, there are also organizational characteristics, such as community 3 (Fig. 3), which show significant commonalities across all the scrutinized task modes and may explain their shared contribution to visual EM and subjective E-AM. Community 3, which brings together ROIs from the DMN, VAN, and SAL, is likely instrumental in the creation and manipulation of mental representations based on environmentally driven attentional and control processes, dynamics that are key to externally cued instances of mental time travel.

Our present findings regarding the neural dynamics correlates of E-AM complement earlier research in an overlapping sample on the stable functional connectivity patterns that distinguish high episodic from semantic SAM scorers (Sheldon et al., 2016). The respective study documented that higher episodic SAM scorers demonstrate stronger intrinsic coupling between medial temporal (MTL) regions and posterior regions implicated in visual perceptual processing. In contrast, higher semantic SAM scorers evidenced stronger functional connectivity between the MTL and frontal regions implicated in categorization. These findings suggest that higher episodic SAM scores may reflect a predisposition towards using visual imagery when accessing the past, while greater semantic SAM scores may indicate a proficiency in organizing information. Extending these findings, the present study provides evidence that the dynamic neural patterns that typify reinstatement of unique spatiotemporal contexts are linked to self-reported E-AM, but not the other SAM subscales. Moreover, broadly consistent with the results of Sheldon et al., that MTL-related functional connectivity patterns suggestive of greater proficiency in categorization are associated with weaker episodic, relative to semantic, memory skills, we show a link between lower self-reported E-AM and a predisposition towards reducing discrete perceptually rich experiences to semanticized representations.

Our present findings lend support to the construct of trait mnemonics, whereby stable, lifelong patterns of encoding information predispose towards engagement in specific mental activities (Palombo et al., 2018b). Whereas high E-AM promotes rich visual re-experiencing of past events that are segregated in consciousness, lower E-AM may be associated with more stable abstract and non-visual representations that generalize across experiences. Accordingly, people with highly superior autobiographical memory (HSAM) have obsessive tendencies that reflect an extreme focus on specific details (LePort et al., 2016), whereas people with severely deficient autobiographical memory (SDAM) show intact learning and daily functioning despite their impaired recollection (Palombo et al., 2015; see also Greenberg and Knowlton, 2014). Beyond these extremes, such biases may yield paradoxical effects, such that those with higher visual EM are more susceptible to visual interference (Sheldon et al., 2017), whereas those with lower E-AM may become resilient to the effects of neurodegenerative disorders affecting EM through the development of cognitive reserve (Stern, 2003; Stern et al., 2004; Fan et al., 2019).

Our present research focused on dispositional variations in the subjective sense that one can revisit one’s past. Individual differences in self-rated E-AM abilities have been shown to be meaningfully related to other cognitive-affective traits, as well as structural and functional brain characteristics (Palombo et al., 2013, 2018a; Sheldon et al., 2016). Such subjective E-AM evaluations, as those assessed by Episodic SAM, are likely to tap distinct aspects of one’s mnemonic experience compared with performance-based measures of E-AM, which assess the quantity of past event fragments that one can recover. For one, differences between subjective and objective E-AM measures may arise due to the fact that the holistic evaluation underlying the former need not equal the sum of the parts indexed by the latter. Second, performance on objective E-AM measures, which index the amount of retrieved event details, can be contaminated by non-EM processes. For example, one can recover details pertaining to a specific event not through mental time travel, but through the repeated use of external aids, such as photographs, diaries, conversations with close others (Cermak and O'Connor, 1983; Rabin et al., 2013). Such alternate routes for retrieving autobiographical details have been used to explain previously demonstrated dissociations between subjective and objective E-AM performance (i.e., recovery of a relatively high amount of episodic details without the accompanying subjective sense of having mentally traveled back to the respective event; Levine et al., 2009). Third, most current measures of objective E-AM focus on the retrieval of a relatively small number of past episodes, which is why performance on these tasks is not necessarily indicative of the capacity to recollect majority of previously experienced events. In our opinion, the SAM-Episodic scale is a useful alternative measure for assessing such stable individual differences in E-AM, albeit from a subjective standpoint.

Our study demonstrates that some of the brain mechanisms that distinguish EM for visual scene sequences from EM for information with significant links to the semantic knowledge base also feed one’s subjective sense of being able to revisit the past. Our findings thus imply that a superior capacity to engage in goal-directed behavior, particularly, to manipulate one’s online mental contents, and a reduced tendency to semanticize perceptually rich mental representations are associated with both visual EM and subjective E-AM skills. Further research is required to determine how this overlap in neurocognitive component processes may support the link between subjective E-AM and visual EM. Evidence for such a link is garnered from recent findings that individuals with higher subjectively rated E-AM show a tighter coupling between oculomotor behavior and objectively assessed E-AM (Armson et al., 2019).

Our present research has several limitations. First, future studies are needed to examine whether spontaneous expression of the neural task modes and sequences, herein investigated, is meaningfully linked to neural connectivity patterns observed during encoding and retrieval of autobiographical memories, as well as with our proposed unfolding of mental processes (e.g., mapping of novel perceptual information onto pre-existing knowledge structures). Second, our SAM sample was primarily composed of younger women, which is why our present results need to be replicated in samples with a balanced gender composition and better lifespan coverage. Third, we used a self-report measure of E-AM because it captures best the experiential aspects of E-AM (i.e., the subjective sense of being able to revisit specific past episodes). As we argued in the Introduction, we propose that this subjective sense-of-self-in-the-past is an emerging property of the state of awareness that typifies retrieval of purely episodic details (i.e., autonoetic consciousness; Tulving, 2002). Our present results suggest that although this mnemonic trait is based on self-report, its supporting brain network architecture is associated with objective performance on EM tasks in a separate sample. Future studies combining performance-based measures of episodic recall with subjective ratings of trait mnemonics in the same sample would be pivotal in shedding further light on the neural mechanisms herein documented.

Finally, to test our main hypotheses, we combined sliding window with graph theoretical analyses of resting state data. The former have been the topic of some controversy. For example, Laumann et al. (2017) provided evidence suggesting that most of the variability associated with resting state connectivity can be accounted for by sampling variability, head motion and sleepiness. Subsequently, it has been pointed out though that Laumann et al. (2017)’s findings are amenable to alternative interpretations, specifically, some that do not exclude the possibility of meaningful fluctuations in resting state connectivity patterns (for an in-depth discussion, see Lurie et al., 2020). Others have also underscored the fact that Laumann et al. (2017)’s results are based on relatively long sliding windows (100 s), which tend to be suboptimal for detecting individual differences in dynamic reorganization patterns and, thus, cannot really speak to the validity of resting state dynamics assessed with shorter time windows (Abrol et al., 2017).

That being said, we do agree that resting state connectivity, particularly when based on shorter time windows, is vulnerable to the influence of confounding factors, including physiological noise and rigid motion. This is why we implemented strict preprocessing procedures for minimizing the impact of such factors (i.e., through CompCor, regression of the 24 motion parameters and their derivatives, use of the summary motion metric in the brain-behavior analyses). Of note, almost 1 h of continuously acquired data went into the main resting state analyses in the HCP sample. The fact that our neural indices based on the sliding window analyses showed reliability values as good as those previously reported for similar graph metrics derived from stable task and resting state connectivity patterns (Braun et al., 2012; Cao et al., 2014), as well as the conceptual replication of the brain-behavior relationships across two independent samples give us confidence in our presently reported results. Nonetheless, future studies using alternate measures of dynamic resting state connectivity would be instrumental in shedding further light on the neural mechanisms herein documented.

In summary, we have provided evidence that individual differences in self-rated E-AM draw on some of the brain mechanisms also implicated in memory for visual scene sequences. These findings support the relationship between subjective mental time travel and visual imagery (Greenberg et al., 2005; D’Argembeau and Van, 2006; Daselaar et al., 2008), specifically, raising the possibility of an association between subjective E-AM and the ability to access temporally ordered mental records of previous experiences. Complementarily, our results imply that perceived difficulties in accessing the past may be traced back to a cognitive style that prioritizes schematic, gist-based information over rich perceptual representations.

10.1523/ENEURO.0531-19.2020.ed1 Extended Data 1 File containing the scripts used in the community detection analyses (“community_louvain.m,” “agreement.m,” “consensus.m [tau = 0, 100 reps]”) and the analyses characterizing similarity in functional brain organization between rest and the various task modes (“normalized_mutual_information.m,” “Max_AMI_multiple.m,” “Count_states.m,” “Count_sequences.m”).Download Extended Data 1, ZIP file.

## Synthesis

Reviewing Editor: Daniela Schiller, Mount Sinai School of Medicine Decisions are customarily a result of the Reviewing Editor and the peer reviewers coming together and discussing their recommendations until a consensus is reached. When revisions are invited, a fact-based synthesis statement explaining their decision and outlining what is needed to prepare a revision will be listed below. The following reviewer(s) agreed to reveal their identity: Christopher Baldassano. Note: If this manuscript was transferred from JNeurosci and a decision was made to accept the manuscript without peer review, a brief statement to this effect will instead be what is listed below.

The study examines novel links between self-rated EM, visual EM, and neural connectivity. The authors have substantially improved this manuscript - the figures are more clear, and more information has been provided about what the data itself looks like.

There are, however, still have several major concerns:

1) The authors have now included a validation experiment as requested, and find that they can classify the task type of a held-out subject’s data with 73% accuracy by comparing to templates derived from the rest of the group. However, rather than using a window of 30s of data as in the primary resting-state experiments, they use all the data from a task run of 220-230s, which is over seven times larger. This is not a valid test of how this classifier would perform on much smaller windows. In the papers the authors cite on lines 338-342, 30s is at the lowest end of the plausible window sizes, and it is not clear whether this is long enough to accurately detect their desired connectivity states. Their rationale for classifying only full runs (as explained in the rebuttal) is not convincing. The authors make two arguments: a) the mapping between mental processes and tasks is not perfectly 1-to-1, which is true but doesn’t seem relevant, since the validation here is attempting to map only from the tasks to the same tasks in a different subject; b) the mental processes of a subject may not be constant across all 30s windows during a task, which is true but seems like it should only lower the ceiling of the best possible decoding accuracy by some amount - if a subject is not “on-task” for a majority of windows within a task, it calls into question whether deriving task-specific templates makes sense in the first place.

2) The way that the authors describe their hypotheses and results is confusing, and it is unclear how these statements relate to the data presented in the text or figures. Here are some of the confusing statements:

a) Lines 31-33: “We propose that superior subjective E-AM and visual EM are both associated with static neural organization principles indicative of greater efficiency in switching between rest and mental states relevant to encoding perceptual information.” On lines 705-706 the authors assert that greater efficiency in switching from rest to task means reduced functional brain reorganization between rest and task. This definition is unclear - there is no measurement of switching efficiency between rest and task in the paper. Also, every window in the resting state data is forced to be classified as one of 4 task-derived states, so it is unclear how this data can tell us how task-like the resting-state data is overall (versus some separate rest-specific state). The interpretation of Fig 5 is that resting-state connectivity looks more similar to a perceptual processing state in high EM subjects, which may or may not have something to do with switching efficiency.

b) Lines 682-686: “we found some support for our hypothesis that individuals with superior subjective E-AM, as well as those with superior visual EM would show organization patterns suggestive of less efficient semantic processing (i.e., reduced similarity in stable functional brain organization between rest and the semantic processing mode, see Figure 5-b).” It is true that there is a negative weight on semantic processing similarity in Fig 5b, but this is only true of the weights and not the loadings (5a) - that is, it is *not* the case that superior EM individuals show less semantic-like connectivity, but that the partial correlation with semantic similarity is negative after accounting for the other variables. It is also not convincing that this canonical weight is statistically significantly less than zero (see below).

3) The authors spend a good deal of time interpreting the individual loadings/weights in Fig 5, but the way that they are identifying which values are significantly different from zero (and drawing the error bars) is not a valid statistical test. They run 10 discovery CCAs by iteratively holding out 1/10 of the data and fitting the CCA to the other 9/10, and the error bars show the range of loadings/weights across those 10 discovery sets. However, these are not 10 independent values - almost all of the data is the same for any pair of discovery sets, so the variability in the loadings/weights is not a fair estimate of the population variability. Since which of these values differ from zero seems important for interpreting the results, the authors should e.g. conduct some type of null hypothesis testing to determine whether these differences are significant.
